# Effect of Blending and Curing Conditions on the Storage Stability of Rubberized Asphalt Binders

**DOI:** 10.3390/ma16030978

**Published:** 2023-01-20

**Authors:** Jihyeon Yun, Shyaamkrishnan Vigneswaran, Moon-Sup Lee, Pangil Choi, Soon-Jae Lee

**Affiliations:** 1 Texas State University, San Marcos, TX 78666, USA; 2 Korea Institute of Civil Engineering and Building Technology, Goyang-si 10223, Republic of Korea; 3 Texas Department of Transportation, Austin, TX 78744, USA

**Keywords:** CRM binder, storage stability, separation index, viscosity, G*/sin *δ*, percentage recovery

## Abstract

Crumb rubber modifier (CRM) binders easily suffer from instability at high temperatures, with many suggestions being developed to evaluate their storage stability. However, much uncertainty around CRM binders still exists regarding the relationship between mixing methods and experiments in order to calculate the separation index. In this study, a laboratory investigation into how CRM binders behave regarding storage stability using different mixing methods and experiments was conducted. The CRM asphalt binder in this study was prepared through a wet mixing process with the addition of 5% and 10% crumb rubber modifier (by weight) at 200 °C. The three main modification methods were method A: high-shear mixing (8000 rpm) for 2 h, method B: low mixing (300 rpm) for 8 h, and method C: high-shear mixing (8000 rpm) for 2 h + low mixing (300 rpm) for 6 h. In addition, the effect of separation index (SI) on storage stability was assessed, measuring viscosity, G*/sin *δ*, and percentage recovery. In general, the results of this study revealed that method C appeared to have the most prominent effect on decreasing the difference between the values of the top and bottom parts; the results for 5% CRM using method C showed that there were no differences among the values for the top, middle, and bottom parts; from the MSCR test, method C was effective in decreasing the difference between the values of the top and bottom parts. It was observed that method C improved storage stability. However, the results for percentage recovery were relatively higher than the separation index when using rotational viscosity and G*/sin *δ*.

## 1. Introduction

Recently, defects in asphalt pavements have been accelerating due to abnormal climate and increased traffic volumes. One common way to improve resistance to these pavement defects is to use modifiers to enhance the physical properties of asphalt binders. Currently, various types of modifiers are being used for this purpose. However, despite the advantages of using modifiers, the factors of inevitable high-temperature transport and storage stability are considered challenges for modified asphalt binders [1].

**Figure 1:**
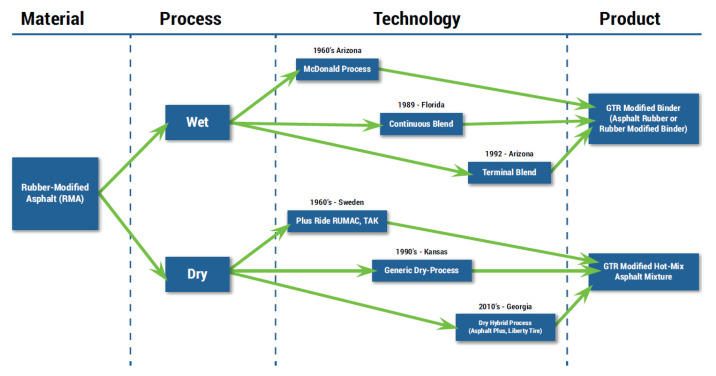
Development of rubber modification process [3].

Crumb rubber modifier (CRM) is one of the most widely used modifiers for asphalt binders that improves engineering properties and offsets environmental problems by using tire waste. Research over the past 20 years has shown that three of the McDonald processes, the traditional wet process, the terminal-blend wet process, and the modern dry process, lead to extended pavement life compared to ordinary asphalt pavements. Moreover, the CRM asphalt mixture can provide performance that is comparable to pavements that are constructed using expensive polymer-modified binders [1,2]. Figure 1 indicates the development of the rubber modification process for asphalt binders.

In particular, the development of the wet process in the United States began in the mid-1960s with Charles McDonald. Although extensive research has been reported in the literature regarding the wet process, the following terms are the most widely used: asphalt rubber (1) is made from at least 15% crumb rubber, with certain additives (by definition) (ASTM D6114). Generally, coarser-sized rubber particles (of about 1.5 mm in size) are used for asphalt rubber production. The interaction between asphalt and crumb rubber occurs at high temperatures ranging from 175–190 °C for approximately 30–60 min. The production and storage of asphalt rubber requires constant agitation. The terminal blends (2) require a similar production process to asphalt rubber. The big difference is that, typically, finer-sized rubber particles (0.600 mm–0.200 mm) are used. Crumb rubber and asphalt are mixed in a mixing tank at a high temperature (175–190 °C) for a minimum of 60 min. The mixture is then stored at elevated temperatures until it is delivered to the worksite. The end blends typically contain about 5–12% rubber and may also contain specialty chemicals or polymers, such as styrene-butadiene-styrene [4]. Recently, even though there has been a study using the analytical hierarchy process method, studies tend to focus on suggesting the best selection and properties of CRM rather than evaluating the methods for modifying CRM and storage stability [5,6]. Moreover, it has been considered that storage instability has still been one of the major issues in wet processes [7].

The instability of CRM asphalt binder at high temperatures presents serious problems for applications. The nonuniform composition and difference in density between asphalt and rubber particles represent the reasons why the rubber particles that are uniformly distributed in the original modified binder tend to fall to the bottom of the tank during storage [8,9]. However, there are various methods to improve the storage stability of CRM asphalt binders, some of which are currently being used in the industry. For example, a recent study showed that the small particle size of CRMs obtained through ambient grounding results in more stable CRM asphalt binders regarding storage stability and that using chemical compounds, such as a mixture of sulfur and styrene-isoprene-styrene (SBS), makes it possible to improve storage stability [10]. In addition, another report showed that nanoclay improved the storage stability of CRM asphalt binder [11,12].

**Figure 2:**
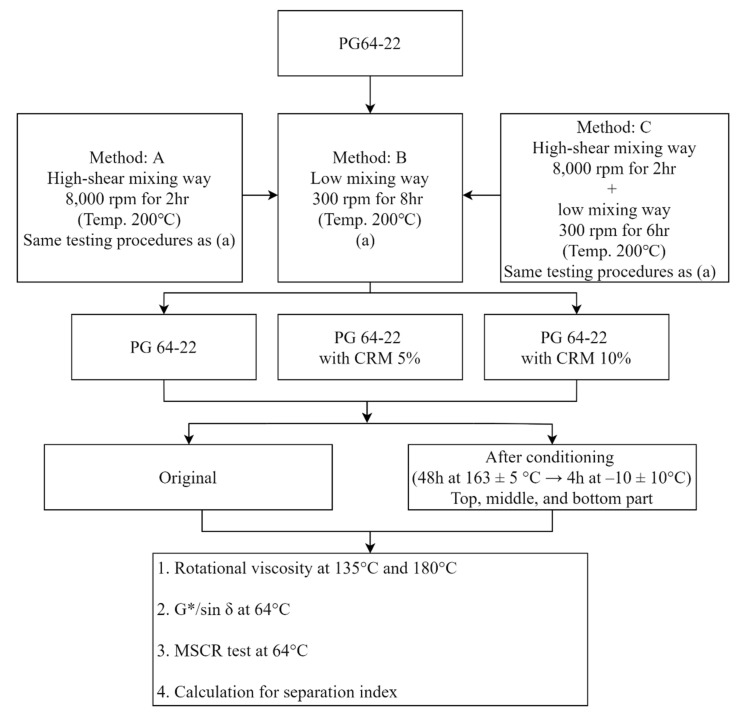
Flow chart of experimental design procedure in this study.

Calculating the separation index (SI) for storage stability has been conducted, according to the ASTM-D7173, to determine whether the modified binders are suitable for storage stability. The calculated value is provided in the results of the softening point (D36/D36M), rheological properties (D7175), and multiple stress creep test (D7405). In particular, the softening point and rheological properties have been comprehensively used to obtain the SI using diverse equations [13,14]. Moreover, a number of researchers reported that the storage stability of modified asphalt binder could be usefully evaluated by means of the softening point and rheological properties [15]. However, much uncertainty still exists about the relationship between the mixing methods and experiments in order to calculate the separation index. Therefore, there are two primary objectives of this study: (1) to evaluate the effect of blending methods on the storage stability of CRM asphalt binders and (2) to present a new opinion on the choice of the SI calculation method by examining how MSCR and rheological properties affect SI. Figure 2 shows the flow chart used for this study.

## 2. Experimental Design

### 2.1. Materials

**Figure 3:**
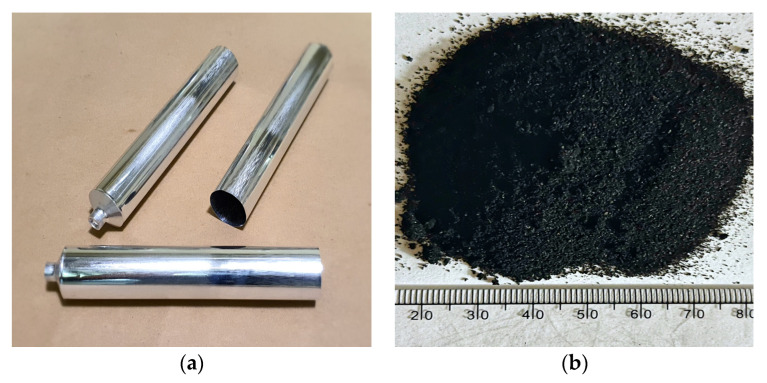
Aluminum tube (**a**) and crumb rubber (**b**).

In this study, base asphalt binder, for which the properties are addressed in Table 1, was used for CRM asphalt binders. The crumb rubber produced by the ambient grinding process was used because it is more effective in producing the CRM binders that are more viscous and less susceptible to rutting and cracking. The CRM particle size is listed in Table 2. The aluminum tube to store the CRM asphalt binder was utilized to evaluate the storage stability based on ASTM D7173. Figure 3 presents the aluminum tube and crumb rubber adopted in this study.

### 2.2. Production and Sampling of CRM Asphalt Binders

The CRM asphalt binder in this study was prepared via a wet mixing process with the addition of 5% and 10% crumb rubber modifier (by weight) at 200 °C. Based on previous research, there are three main modification methods [16,17,18,19,20]:

Method A: High-shear mixing (8000 rpm) for 2 h;

Method B: Low mixing (300 rpm) for 8 h;

Method C: High-shear mixing (8000 rpm) for 2 h + low mixing (300 rpm) for 6 h.

**Figure 4:**
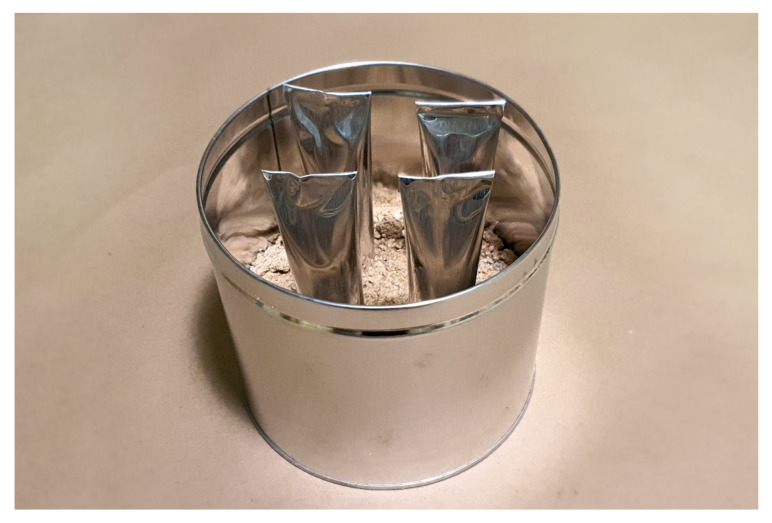
The vertically positioned tube.

The original CRM asphalt sample was immediately prepared for the viscoelastic evaluation after the blending, and then the other remains of this binder were carefully stirred and filled with 50 ± 0.5 g into the vertically held tube to become conditioned in an oven at 163 ± 5 °C for 48 ± 1 h (Figure 4). At the end of this conditioning, the tube was moved to the freezer at –10 ± 10 °C in order to thoroughly make the binder hard for at least 4 h, constantly keeping them vertical at all times. After solidifying the CRM binder, each tube was cut into three parts of almost equal length, which were then placed into an oven at 163 ± 5 °C for up to 30 min to make them fully fluid for removing the aluminum tube. Finally, the binder specimens for each evaluation were prepared.

### 2.3. Binder Evaluation

#### 2.3.1. Rotational Viscosity

**Figure 5:**
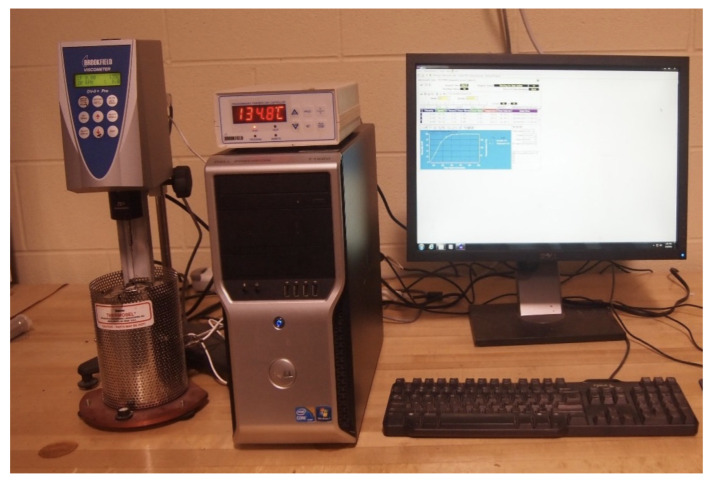
The rotational viscometer.

The rotational viscosity result is a factor used for evaluating the workability (from the production to the compaction of asphalt mixture) of all binders in order to evaluate the viscous property according to AASHTO T 316, which were measured with a Brookfield rotational viscometer at the temperatures of 135 °C and 180 °C using the 27 cylindrical spindles and a constant velocity of 20 rpm with 10.5 g of CRM binder. The testing time to obtain results was applied at 20 min for all samples. The Brookfield rotational viscometer used in this study is shown in Figure 5.

#### 2.3.2. Rheological Property and MSCR

**Figure 6:**
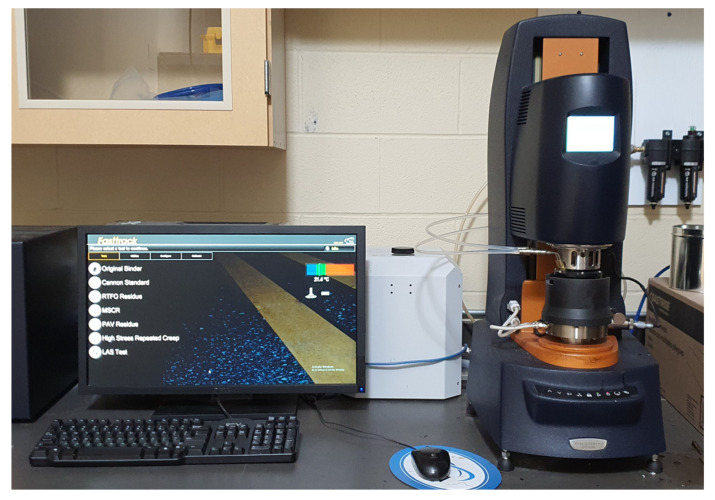
The dynamic shear rheometer.

This section, based on a dynamic shear rheometer (DSR) (Figure 6), analyzes and classifies the viscoelastic properties of CRM asphalt binder from two standards: (1) G*/sin *δ* by ASTM D7175 (2) % rec by ASTM D7405. The first evaluation was conducted based on the results from the complex shear modulus (G*) and the sin (*δ*) at 64 °C. For the second evaluation, the MSCR test was performed to measure % rec by applying a load of 0.1 kPa and 3.2 kPa at the same temperature as the first evaluation.

#### 2.3.3. Separation Index (SI)

In this study, the CRM asphalt binder was analyzed to obtain the SI with the results of the top and bottom parts. First of all, a viscosity property at a high temperature was used to calculate the SI based on Equation (1), where (Viscosity)_max_ indicates the higher value between the top and bottom parts and (Viscosity)_avg_ is the average value for both parts. As per the above calculation for SI, G*/sin *δ* was used to evaluate the separation ratio via Equation (2) [21]. Moreover, % rec was adopted as a value to calculate the SI using Equation (3). Finally, the three results of the SI were compared with each other.

(1) $$Separation\;index=\frac{{\left(\mathrm{Viscosity}\right)}_{\max}-{\left(\mathrm{Visosity}\right)}_{\mathrm{avg}}}{{\left(\mathrm{Viscosity}\right)}_{\mathrm{avg}}}\;$$

(2) $$Separation\;index=\frac{{\left(G*/\sin\delta \right)}_{\max}-{\left(G*/\sin\delta \right)}_{\mathrm{avg}}}{{\left(G*/\sin\delta \right)}_{\mathrm{avg}}}\;$$

(3) $$Separation\;index=\frac{{\left(\%\;\mathrm{rec}\right)}_{\max}-{\left(\%\;\mathrm{rec}\right)}_{\mathrm{avg}}}{{\left(\%\;\mathrm{rec}\right)}_{\mathrm{avg}}}\;$$

## 3. Results and Discussions

### 3.1. Rotational Viscosity

**Figure 7:**
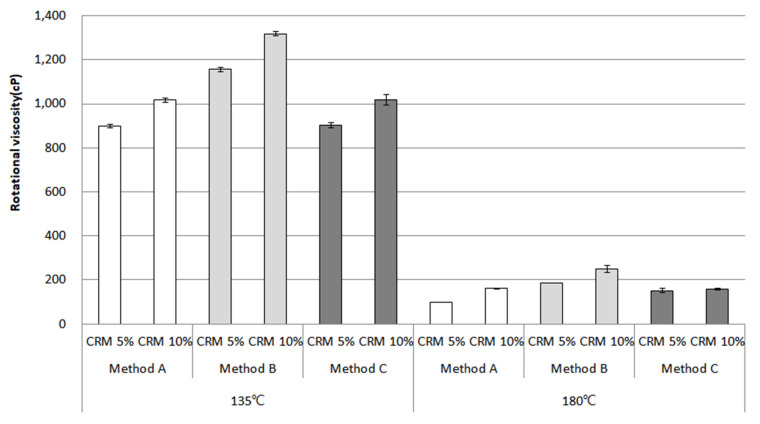
Rotational viscosity of the CRM asphalt binders in each mixing method for the original condition.

Rotational viscosity (RV) is an important component in the evaluation of the workability from production to the compaction of the asphalt mixture. In order to give an example, high viscosity can affect the density of the asphalt mixture at the work site, making it difficult to have optimum air voids and voids in the mineral aggregate. In this study, the RV of the CRM asphalt binders was assessed at temperatures of 135 °C and 180 °C. Figure 7 shows the RV results for the original condition in each mixing method. Overall, the RV increased slightly, as expected, from the addition of 5% to 10% CRM and both contents showed a relatively high RV between methods A and B. On the other hand, the RV for method C remained low. This result is considered due to the fact that, during the high-shear mixing for 2 h, the CRM particles are in a smooth and homogeneous state and that the CRM particles are digested and dissolved over a relatively long mixing time, resulting in lower viscosity for method C.

**Figure 8:**
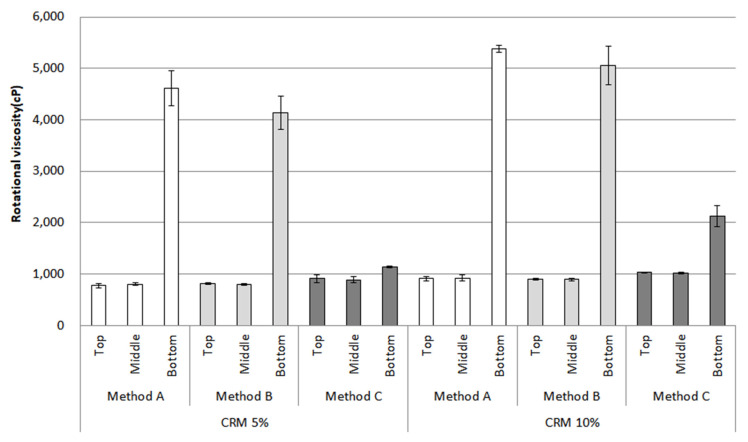
Rotational viscosity (135 °C) of the CRM asphalt binders in each mixing method for the top, middle, and bottom parts after conditioning.

The RV after conditioning is shown in Figure 8. This bar chart depicts the results of the top, middle, and bottom parts at a testing temperature of 135 °C. The RV evaluated by methods A and B increased sharply for the top to bottom parts, resulting in roughly four times in CRM 5% and five times in CRM 10%; meanwhile, for method C, the RV for CRM 5% remained steady. Even though the RV for CRM 10% in method C also increased, this indicates an insignificant rise compared to methods A and B.

**Figure 9:**
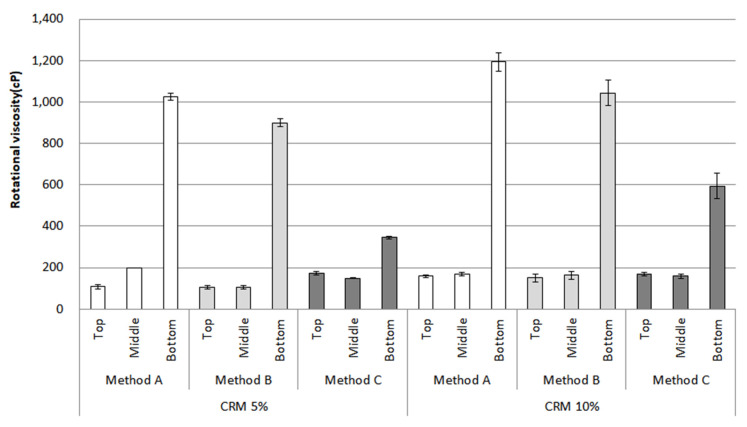
Rotational viscosity (180 °C) of the CRM asphalt binders in each mixing method for the top, middle, and bottom parts after conditioning.

The overall tendency for the RV in Figure 9 increases at 180 °C from the top to bottom parts is similarly shown for the results at 135 °C. In particular, method C showed a relatively low increase compared to other mixing methods. As discussed in the results for the original condition, the main reason for this trend (related to the low increase in the RV) in method C is influenced by the mixing method. Moreover, as for the RV difference between the top and bottom parts, since this indicates storage instability for the asphalt binders, it is confirmed that designing a mixing method for CRM asphalt binders is important for storage stability.

The approach to statistical significance (α = 0.05) adopted for this study was to use a one-way analysis of variance (ANOVA) for the data before and after conditioning. The significant differences were mostly witnessed between the top and bottom parts among all the mixing methods at both temperatures of 135 °C and 180 °C (Table 3). The result for 5% CRM using method C showed an insignificant difference at 135 °C. However, there were significant differences between the top and bottom parts for all data at 180 °C. Based on the results of the bar charts and the statistical results, The most prominent method to enhance the storage stability of CRM asphalt binders was considered to be method C (using both high-shear mixing and low-speed mixing methods) due to the mechanism by which the interaction between the CRMs and binders occurs.

### 3.2. Rheological Property

**Figure 10:**
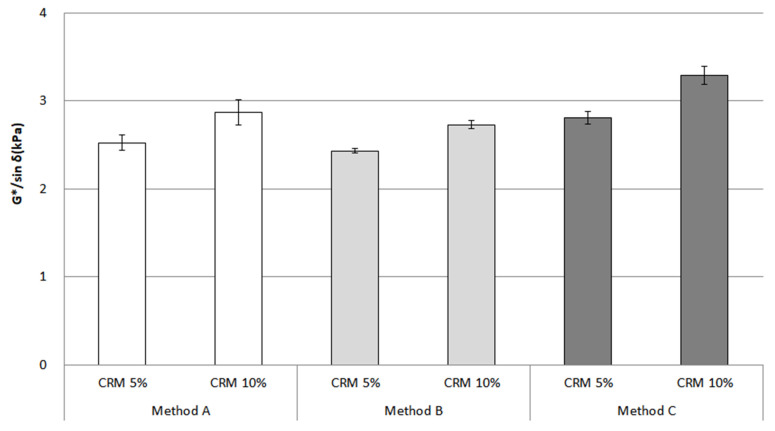
G*/sin *δ* of the CRM asphalt binders for each mixing method in the original condition.

The rheological properties (dynamic shear rheometer (DSR)) represent the most common factor for evaluating storage stability. In this study, G*/sin *δ* was adopted to test the rheological properties of the CRM asphalt binders. The original condition (prepared immediately after modifying the CRM asphalt binders) was measured at 64 °C (Figure 10). As expected, an increasing trend for G*/sin *δ* among all the mixing methods was identified.

**Figure 11:**
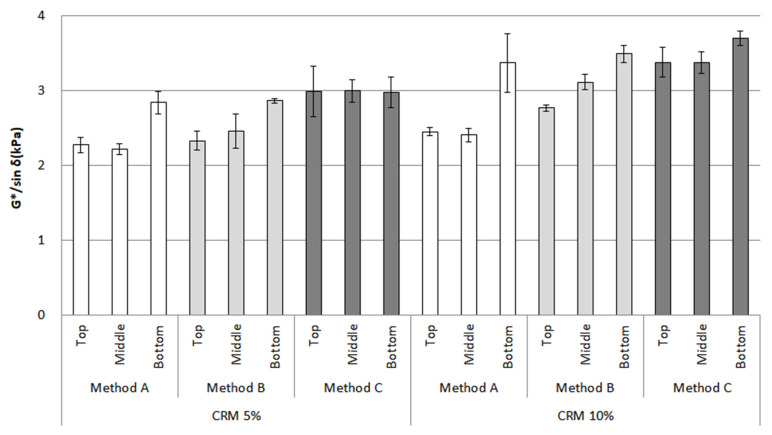
G*/sin *δ* of the CRM asphalt binders for each mixing method for the top, middle, and bottom parts after conditioning.

Figure 11 shows that the CRM asphalt binders, after conditioning, appeared to have an increasing trend for G*/sin *δ* regarding the top and bottom parts; in particular, methods A and B show a relatively big difference among all the CRM contents and mixing methods. On the contrary, method C, by far, saw the lowest difference between the top and bottom parts, even with 5% CRM; this content remained relatively stable and unchanged among the top, middle, and bottom parts. As previously described in the RV section, method C has a more positive effect on storage stability for CRM asphalt binder than methods A and B.

An ANOVA was applied to analyze the statistical significance of the G*/sin *δ* value among the original condition, top, and bottom parts for 5% and 10% CRM (Table 4). This investigation shows that between the top and bottom parts, there were mostly significant influences on the G*/sin *δ* value of CRM asphalt binders. However, this result indicated no significant difference for only 5% CRM. When considering the bar chart for the rheological properties above, the mixing conditions could affect storage stability; in particular, 5% CRM was found to be an optimum content to enhance storage stability. Moreover, these results correspond with the bar chart for G*/sin *δ*.

### 3.3. MSCR Test

**Figure 12:**
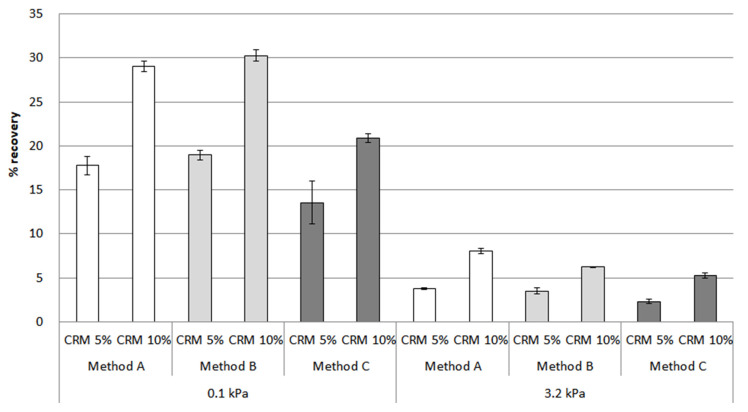
The percentage recovery of the CRM asphalt binders for each mixing method under the original condition.

The MSCR test is considered an alternative test to obtain factors for evaluating the storage stability of the modified asphalt binders. Based on loadings of 0.1 kPa and 3.2 kPa, the percentage recovery for the CRM asphalt binders was measured at 64 °C. Figure 12 shows the results of the original condition for each CRM content and mixing method. Throughout this bar chart, it is clear that the increasing trend for percentage recovery was seen with the addition of CRM content, as expected. Regarding the results at 0.1 kPa, the value of the percentage recovery was higher than the results at 3.2 kPa. In particular, the gap in percentage recovery at 0.1 kPa is around two times higher than at 3.2 kPa.

**Figure 13:**
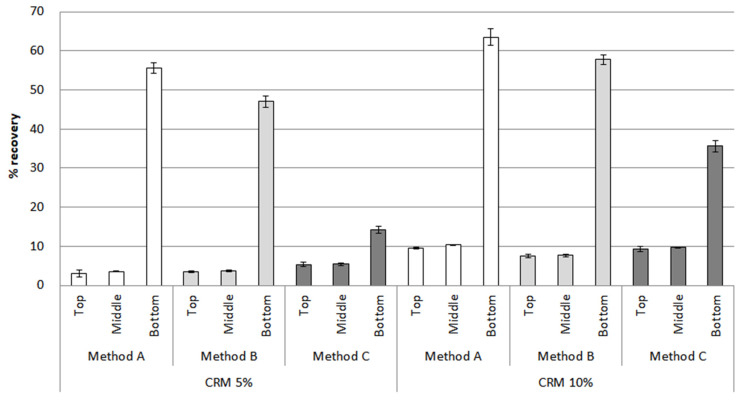
The percentage recovery (0.1 kPa) of the CRM asphalt binders for each mixing method in the top, middle, and bottom parts, after conditioning.

**Figure 14:**
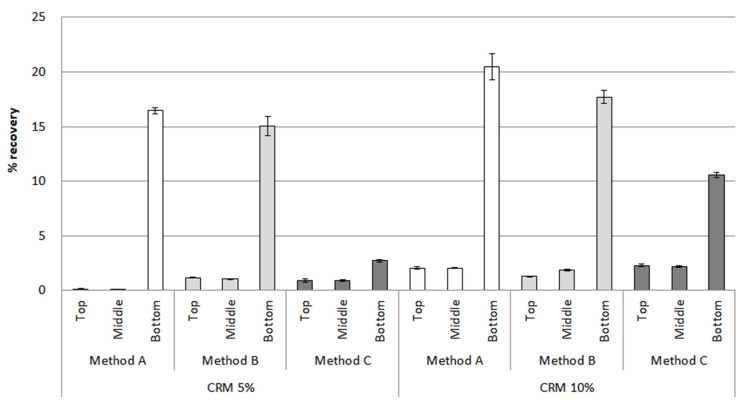
The percentage recovery (3.2 kPa) of the CRM asphalt binders for each mixing method in the top, middle, and bottom parts, after conditioning.

After the conditioning of the CRM asphalt binders in Figure 13, method A showed the biggest difference between the top and bottom parts for 5% and 10% CRM, reaching peaks of 55% and 65% in the bottom parts, respectively. The percentage recovery of method B was relatively low between the top and bottom parts, with the results of bottom parts accounting for 48% in 5% CRM and 58% in 10% CRM, compared to method A, in which there still remained a big difference. In the case of method C, the overall percentage recovery value dropped compared to other methods; in particular, the result for 5% CRM showed its lowest point, at around 15% in the bottom parts. Moreover, the difference between the percentage recovery of the top and bottom parts is noticeably low in method C, suggesting method C can be effective in enhancing the storage stability of the CRM asphalt binders. For the percentage recovery results under 3.2 kPa in Figure 14, the percentage recovery was lower than when applying a load of 0.1 kPa due to the application of a high load. In addition, the difference in the percentage recovery results of the top and bottom parts was larger than that of the 0.1 kPa load. However, the overall trends for the 0.1 kPa and 3.2 kPa results were similar. As mentioned in the RV section above, this MSCR result among the parts is drawn due to the CRM particles reacting differently with the binder according to the mixing method.

The statistical significance of the change in percentage recovery for the CRM asphalt binders was evaluated using ANOVA, comparing each part among the different mixing methods. In terms of a comparison between the top and bottom parts, a significant difference was observed for all mixing methods and both loads (of 0.1 kPa and 3.2 kPa) in Table 5. This result means that the CRM particles were fully separated from the asphalt binder, remaining in the bottom parts. When considering the evaluation methods for storage stability, even though the rheological properties and MSCR were mainly introduced from ASTM7173, the results showed that they behave differently when evaluating storage stability.

### 3.4. Storage Stability Results

**Figure 15:**
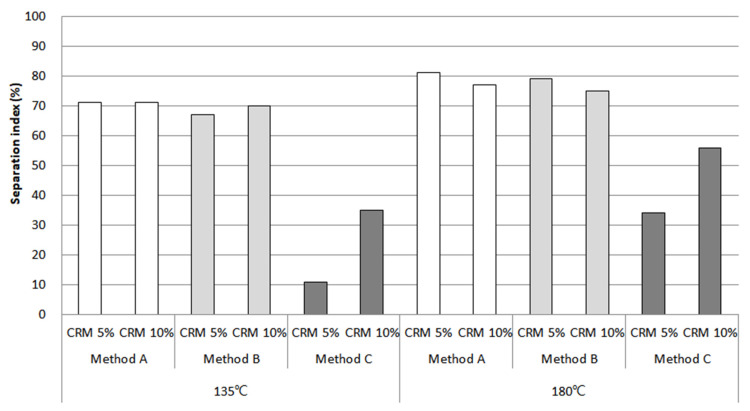
Separation index from rotational viscosity.

**Figure 16:**
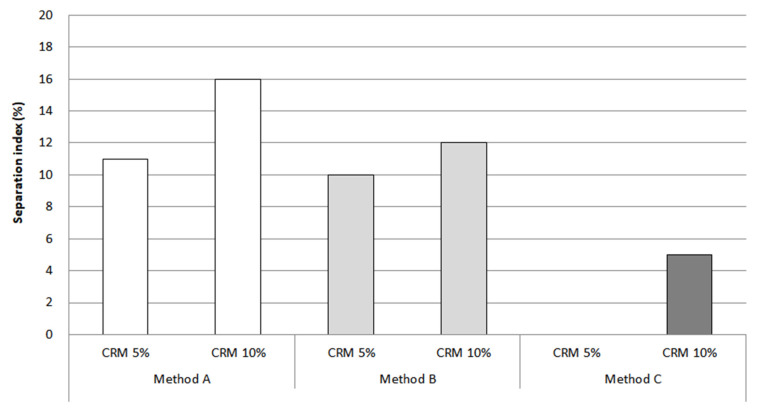
Separation index from G*/sin *δ*.

**Figure 17:**
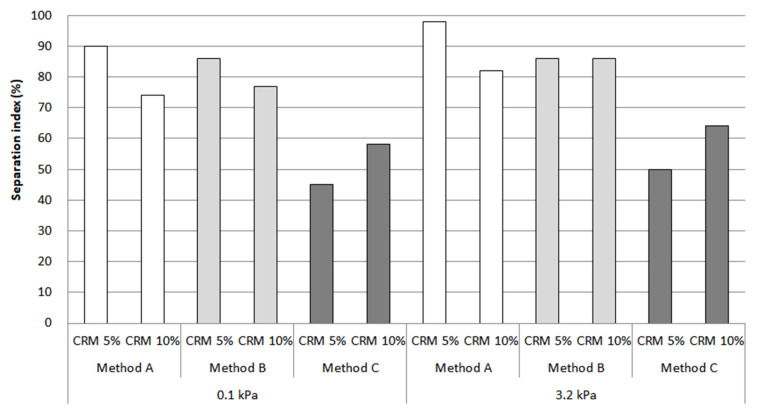
Separation index from percentage recovery.

In this study, the storage stability results were based on data from rotational viscosity, rheological properties, and MSCR. Although extensive research was carried out (Figure 15, Figure 16 and Figure 17), only method C showed the most prominent effect on storage stability for a CRM asphalt binder. In particular, the separation index for both contents using rotational viscosity at 135 °C dropped sharply to a low of around 10% in 5% CRM and 35% in 10% CRM. In the case of the results at 180 °C, the result indicated a decreasing trend to 35% in 5% CRM and 55% in 10% CRM. The main reason is that method C positively affects the digestion of the CRM particles with high-shear mixing and then allows them to be fully digested and better dissolved into the asphalt binder with low-shear stirring.

When utilizing the G*/sin *δ* value for the evaluation of storage stability, the result for 5% CRM revealed that method C improved storage stability, and the separation index remained at 0%. For 10% CRM, the index was about 5%, but this case was still effective with a value of more than two-fold lower than methods A and B. As mentioned in the results for the separation index of the rotational viscosity, method C was shown to improve the storage stability of a CRM asphalt binder.

The separation index results using percentage recovery showed that method C was still effective for storage stability, compared to methods A and B. However, the result in Figure 17 is relatively higher than the separation index using rotational viscosity and G*/sin *δ*. This is because the MSCR test was evaluated using a relatively higher load than that of G*/sin *δ*, which could detect CRM particles that were still not dispersed and absorbed, even including very fine CRM particles. This study indicates a need to consider the various viewpoints for selecting factors in order to properly evaluate storage stability.

## 4. Conclusions

In order to investigate the storage stability of CRM asphalt binders, both 5% and 10% CRM contents were evaluated before and after conditioning using three mixing methods while adopting the factors of rotational viscosity, G*/sin *δ*, and percentage recovery. Based on the results of this study, the following conclusions were drawn.

1. Adding CRM resulted in an increasing trend for rotational viscosity at temperatures of 135 °C and 180 °C, as per expectations. Method C appeared to have the most prominent effect when decreasing the difference between the values of the top and bottom parts, while methods A and B resulted in high differences;
2. An increasing trend for G*/sin *δ* was witnessed as the CRM was added. The result for 5% CRM using method C showed that there were no differences among the values of the top, middle, and bottom parts, suggesting method C can be effective for storage stability. By contrast, methods A and B remained high regarding the differences between the top and bottom parts, as per the rotational viscosity results;
3. In the MSCR test, percentage recovery was measured, resulting in an increasing trend with increasing CRM content. However, even though method C allowed for an effective decrease in the difference between the values of the top and bottom parts, all mixing methods showed relatively big differences between these parts when compared to the data from the rotational viscosity and rheological properties;
4. For the storage stability of CRM asphalt binder, it was observed that method C improved storage stability; in particular, when using G*/sin *δ* of 5% CRM, the results indicated a 0% separation index. On the other hand, the results for percentage recovery were relatively higher than the separation index using rotational viscosity and G*/sin *δ*;
5. In this study, it was confirmed that designing a mixing method for CRM asphalt binders is important for storage stability. The main reason is that the mixing methods can affect the digestion of the CRM particles, which allows them to be dissolved into the asphalt binder. Moreover, since the SI results vary depending on the experimental method standards, without taking this into consideration, the results do not present a correct conclusion about storage stability;
6. Much uncertainty still exists about the relationship among modifiers, mixing methods, and the factors for calculating the separation index. Comprehensive research is required to suggest practical methods for increasing storage stability.

## Figures and Tables

**Table 1:** Properties of base asphalt binder (PG 64–22).

	Test Properties	Test Result
Original binder	Viscosity @ 135 °C (cP)	538
	G*/sin *δ* @ 64 °C (kPa)	1.38
RTFO aged binder	G*/sin *δ* @ 64 °C (kPa)	3.82
RTFO + PAV aged binder	G*/sin *δ* @ 25 °C (kPa)	4402
	Stiffness @ −12 °C (MPa)	205
	m-value @ −12 °C	0.323

**Table 2:** Gradation of Crumb rubber modifier adopted in this study.

Sieve Number (μm)	% Cumulative Passed
30 (600)	100
50 (300)	57.7
100 (150)	14.2
200 (75)	0.0

**Table 3:** Statistical analysis results for the rotational viscosity of the CRM binders as a function of the original, top, and bottom parts (α = 0.05).

			135 °C																	
			CRM 5%									CRM 10%								
			Method A			Method B			Method C			Method A			Method B			Method C		
			O	T	B	O	T	B	O	T	B	O	T	B	O	T	B	O	T	B
CRM 5%	Method A	O	-	N	S	N	N	S	N	N	N	N	N	S	N	N	S	N	N	S
		T		-	S	S	N	S	N	N	S	N	N	S	S	N	S	N	N	S
		B			-	S	S	**S**	S	S	S	S	S	S	S	S	S	S	S	S
	Method B	O				-	S	S	N	N	N	N	N	S	N	N	S	N	N	S
		T					-	S	N	N	S	N	N	S	S	N	S	N	N	S
		B						-	S	S	S	S	S	S	S	S	S	S	S	S
	Method C	O							-	N	N	N	N	S	S	N	S	N	N	S
		T								-	N	N	N	S	S	N	S	N	N	S
		B									-	N	N	S	N	N	S	N	N	S
CRM 10%	Method A	O										-	N	S	N	N	S	N	N	S
		T											-	S	S	N	S	N	N	S
		B												-	S	S	S	S	S	S
	Method B	O													-	S	S	N	N	S
		T														-	S	N	N	S
		B															-	S	S	S
	Method C	O																-	N	S
		T																	-	S
		B																		-
			180 °C																	
CRM 5%	Method A	O	-	S	S	S	N	S	N	S	S	S	S	S	S	N	S	S	S	S
		T		-	S	S	S	S	S	S	S	S	S	S	S	S	S	S	S	S
		B			-	S	S	**S**	S	S	S	S	S	S	S	S	N	S	S	S
	Method B	O				-	S	S	N	N	S	N	N	S	S	N	S	N	N	S
		T					-	S	N	S	S	S	N	S	S	N	S	N	S	S
		B						-	S	S	S	S	S	S	S	S	S	S	S	S
	Method C	O							-	N	S	N	N	S	S	N	S	N	N	S
		T								-	S	N	N	S	S	N	S	N	N	S
		B									-	S	S	S	S	S	S	S	S	S
CRM 10%	Method A	O										-	N	S	S	N	S	N	N	S
		T											-	S	S	N	S	N	N	S
		B												-	S	S	S	S	S	S
	Method B	O													-	S	S	S	S	S
		T														-	S	N	N	S
		B															-	S	S	S
	Method C	O																-	N	S
		T																	-	S
		B																		-

O: Original, T: Top part, B: Bottom part. N: nonsignificant, S: significant.

**Table 4:** Statistical analysis results of the G*/sin *δ* of the CRM binders as a function of the original, top, and bottom parts (α = 0.05).

			G*/sin *δ*																	
			CRM 5%									CRM 10%								
			Method A			Method B			Method C			Method A			Method B			Method C		
			O	T	B	O	T	B	O	T	B	O	T	B	O	T	B	O	T	B
CRM 5%	Method A	O	-	N	S	N	N	S	S	S	S	S	N	S	N	N	S	S	S	S
		T		-	S	N	N	S	S	S	S	S	N	S	S	S	S	S	S	S
		B			-	S	S	**N**	N	N	N	N	S	S	N	N	S	S	S	S
	Method B	O				-	N	S	S	S	S	S	N	S	S	S	S	S	S	S
		T					-	S	S	S	S	S	N	S	S	S	S	S	S	S
		B						-	N	N	N	N	S	S	N	N	S	S	S	S
	Method C	O							-	N	N	N	S	S	N	N	S	S	S	S
		T								-	N	N	S	S	N	N	S	S	S	S
		B									-	N	S	S	N	N	S	S	S	S
CRM 10%	Method A	O										-	S	S	N	N	S	S	S	S
		T											-	S	S	S	S	S	S	S
		B												-	S	S	N	N	N	S
	Method B	O													-	N	S	S	S	S
		T														-	S	S	S	S
		B															-	N	N	N
	Method C	O																-	N	S
		T																	-	S
		B																		-

O: Original, T: Top part, B: Bottom part. N: non-significant, S: significant.

**Table 5:** Statistical analysis results of percentage recovery of the CRM binders as a function of the original, top, and bottom parts (α = 0.05).

			0.1 kPa																	
			CRM 5%									CRM 10%								
			Method A			Method B			Method C			Method A			Method B			Method C		
			O	T	B	O	T	B	O	T	B	O	T	B	O	T	B	O	T	B
CRM 5%	Method A	O	-	S	S	N	S	S	S	S	S	S	S	S	S	S	S	S	S	S
		T		-	S	S	N	S	S	S	S	S	S	S	S	S	S	S	S	S
		B			-	S	S	**S**	S	S	S	S	S	S	S	S	S	S	S	S
	Method B	O				-	S	S	S	S	S	S	S	S	S	S	S	S	S	S
		T					-	S	S	S	S	S	S	S	S	S	S	S	S	S
		B						-	S	S	S	S	S	S	S	S	S	S	S	S
	Method C	O							-	S	N	S	S	S	S	S	S	S	S	S
		T								-	S	S	S	S	S	S	S	S	S	S
		B									-	S	S	S	S	S	S	S	S	S
CRM 10%	Method A	O										-	S	S	N	S	S	S	S	S
		T											-	S	S	S	S	S	S	S
		B												-	S	S	S	S	S	S
	Method B	O													-	S	S	S	S	S
		T														-	S	S	S	S
		B															-	S	S	S
	Method C	O																-	S	S
		T																	-	S
		B																		-
			3.2 kPa																	
CRM 5%	Method A	O	-	S	S	N	S	S	S	S	S	S	S	S	S	S	S	S	S	S
		T		-	S	S	S	S	S	S	S	S	S	S	S	S	S	S	S	S
		B			-	S	S	**S**	S	S	S	S	S	S	S	S	S	S	S	S
	Method B	O				-	S	S	S	S	S	S	S	S	S	S	S	S	S	S
		T					-	S	S	N	S	S	S	S	S	N	S	S	S	S
		B						-	S	S	S	S	S	S	S	S	S	S	S	S
	Method C	O							-	S	N	S	N	S	S	S	S	S	N	S
		T								-	S	S	S	S	S	N	S	S	S	S
		B									-	S	N	S	S	S	S	S	S	N
CRM 10%	Method A	O										-	S	S	S	S	S	S	S	S
		T											-	S	S	S	S	S	N	S
		B												-	S	S	S	S	S	S
	Method B	O													-	S	S	S	S	S
		T														-	S	S	S	S
		B															-	S	S	S
	Method C	O																-	S	S
		T																	-	S
		B																		-

O: Original, T: Top part, B: Bottom part. N: nonsignificant, S: significant.

## Data Availability

The data used to support the findings of this study are included within the article.
